# Unexpected Salt/Cocrystal Polymorphism of the Ketoprofen–Lysine System: Discovery of a New Ketoprofen–l-Lysine Salt Polymorph with Different Physicochemical and Pharmacokinetic Properties

**DOI:** 10.3390/ph14060555

**Published:** 2021-06-10

**Authors:** Andrea Aramini, Gianluca Bianchini, Samuele Lillini, Simone Bordignon, Mara Tomassetti, Rubina Novelli, Simone Mattioli, Larisa Lvova, Roberto Paolesse, Michele Remo Chierotti, Marcello Allegretti

**Affiliations:** 1 Research and Early Development, Dompé Farmaceutici S.p.A., 67100 L’Aquila, Italy; gianluca.bianchini@dompe.com (G.B.); marcello.allegretti@dompe.com (M.A.); 2 Research and Early Development, Dompé Farmaceutici S.p.A., 80131 Napoli, Italy; samuele.lillini@dompe.com (S.L.); mara.tomassetti@dompe.com (M.T.); simone.mattioli@shuttle-tech.com (S.M.); 3 IMAST S.c.a.r.l.—Technological District on Engineering of Polymeric and Composite Materials and Structures, 80133 Napoli, Italy; 4 Department of Chemistry and NIS Centre, University of Torino, 10125 Torino, Italy; simone.bordignon@unito.it (S.B.); michele.chierotti@unito.it (M.R.C.); 5 Research and Early Development, Dompé Farmaceutici S.p.A., 20122 Milano, Italy; rubina.novelli@dompe.com; 6 Department of Chemical Science and Technology, University of Rome Tor Vergata, 00133 Rome, Italy; larisa.lvova@uniroma2.it (L.L.); roberto.paolesse@uniroma2.it (R.P.)

**Keywords:** ketoprofen–l-lysine salt, cocrystal, salt, polymorphism, faster-release formulation

## Abstract

Ketoprofen–l-lysine salt (KLS) is a widely used nonsteroidal anti-inflammatory drug. Here, we studied deeply the solid-state characteristics of KLS to possibly identify new polymorphic drugs. Conducting a polymorph screening study and combining conventional techniques with solid-state nuclear magnetic resonance, we identified, for the first time, a salt/cocrystal polymorphism of the ketoprofen (KET)–lysine (LYS) system, with the cocrystal, KET–LYS polymorph 1 (P1), being representative of commercial KLS, and the salt, KET–LYS polymorph 2 (P2), being a new polymorphic form of KLS. Interestingly, in vivo pharmacokinetics showed that the salt polymorph has significantly higher absorption and, thus, different pharmacokinetics compared to commercial KLS (cocrystal), laying the basis for the development of faster-release/acting KLS formulations. Moreover, intrinsic dissolution rate (IDR) and electronic tongue analyses showed that the salt has a higher IDR, a more bitter taste, and a different sensorial kinetics compared to the cocrystal, suggesting that different coating/flavoring processes should be envisioned for the new compound. Thus, the new KLS polymorphic form with its different physicochemical and pharmacokinetic characteristics can open the way to the development of a new KET–LYS polymorph drug that can emphasize the properties of commercial KLS for the treatment of acute inflammatory and painful conditions.

## 1. Introduction

During the last decade, cocrystallization has emerged and established itself in the field of pharmaceutical solid-state chemistry as an advanced and valuable method to modify the physicochemical characteristics of drugs [1]. Like pharmaceutical salts, cocrystals are multi-component crystalline solid materials formed by two or more molecules, of which at least one is an active pharmaceutical ingredient (API) that is combined with generally recognized as safe (GRAS), organic molecules used as cocrystallizing agents, or coformers [2]. Pharmaceutical salts and cocrystals, thus, preserve the intrinsic activities of the APIs, while their physicochemical properties can be tailored systematically by varying the coformers [3]. Contrary to salts, however, in which the molecules within the crystal lattice predominantly interact through ion pairing [4], in cocrystals, the components are combined via other noncovalent interactions (e.g., hydrogen bonds, van der Waals forces, π-stacking, and electrostatic interactions) in a definite stoichiometric ratio [5,6]. Thus, particularly when hydrogen bonds (HBs) are involved, cocrystals can be distinguished from salts because, in the latter, a complete proton transfer occurs along the axis of the HB interaction between the API and the molecular partner, whereas, in cocrystals, a neutral adduct is formed by the components [7].

Most commonly, when a specific pair of molecules is combined with the same stoichiometry and at the same temperature, the outcome is either a salt (ionic) or a cocrystal (neutral) [8]. However, to predict whether the outcome will be one or the other can be difficult. For this reason, a “p*K_a_* rule” has been proposed to provide, at least to some extent, reliable indications about the possibility of obtaining a salt and vice versa. Accordingly, a larger Δp*K_a_* (i.e., p*K_a_* (protonated base) − p*K_a_* (acid)) leads to a greater chance of obtaining a salt and vice versa [9,10,11], while the probability can also be quantitatively estimated with formulas as follows (for the salt and the cocrystal, respectively) [10]:

(Pobs(AB)/% = −17Δp*K_a_* + 72 and Pobs(A^−^ B^+^)/% = 17Δp*K_a_* + 28 (1)

These formulas identify a region of uncertainty around Δp*K_a_* = 1, wherein the chances to get a salt or a cocrystal are very similar. In this region usually fall the rare cases in which the same chemical species can give rise to both a cocrystal and a salt (salt/cocrystal polymorphism), which have the same composition and stoichiometry at the same temperature, depending on the crystallization protocol. Stainton et al. were the first to report a cocrystal and a salt of the same chemical composition (isonicotinamide and citric acid [12]) and, subsequently, other cases of salt/cocrystal polymorphisms were found for β-alanine and dl-tartaric acid [13,14], sulfamethazine and saccharine [15], dinitrobenzoic acid and haloanilines [16], and ethionamide and salicylic acid [8]. Some of these systems were analyzed for their thermodynamic stability [14], moisture sorption and compaction properties [15], or dissolution rate [8], and cocrystals resulted in having superior or similar properties compared to those of the polymorphic salts. Only a limited number of these studies, however, compare head-to-head salt and cocrystal polymorphic forms originated from the same chemical species in terms of physicochemical properties and in vivo pharmacokinetic and pharmacodynamic properties, leaving still open the question about the actual impact that the physicochemical difference between salt and cocrystal polymorphic forms can have on the clinical performance/pharmacological properties of a drug. Answering this question is of particular interest, not only because of the regulatory and legal implications of intellectual property rights related to the discovery of a new polymorph [17], but most importantly because it could lead to the development of a drug that, while maintaining the same chemical composition of the polymorphic one, can have different pharmacokinetic and pharmacological characteristics. According to its physicochemical and pharmacokinetic properties, the polymorphic form may allow for the development of different formulations [18], which can be characterized, for example, by a slower or faster drug release in the blood; on the other hand, depending on its taste, a different coating process or flavor can be used to mask the drug taste, thus possibly affecting drug manufacturing costs and patient compliance [19]. Lastly, on the basis of all the pharmacokinetic and physicochemical characteristics of the new polymorph, new therapeutic indications can potentially be hypothesized.

**Scheme 1:**
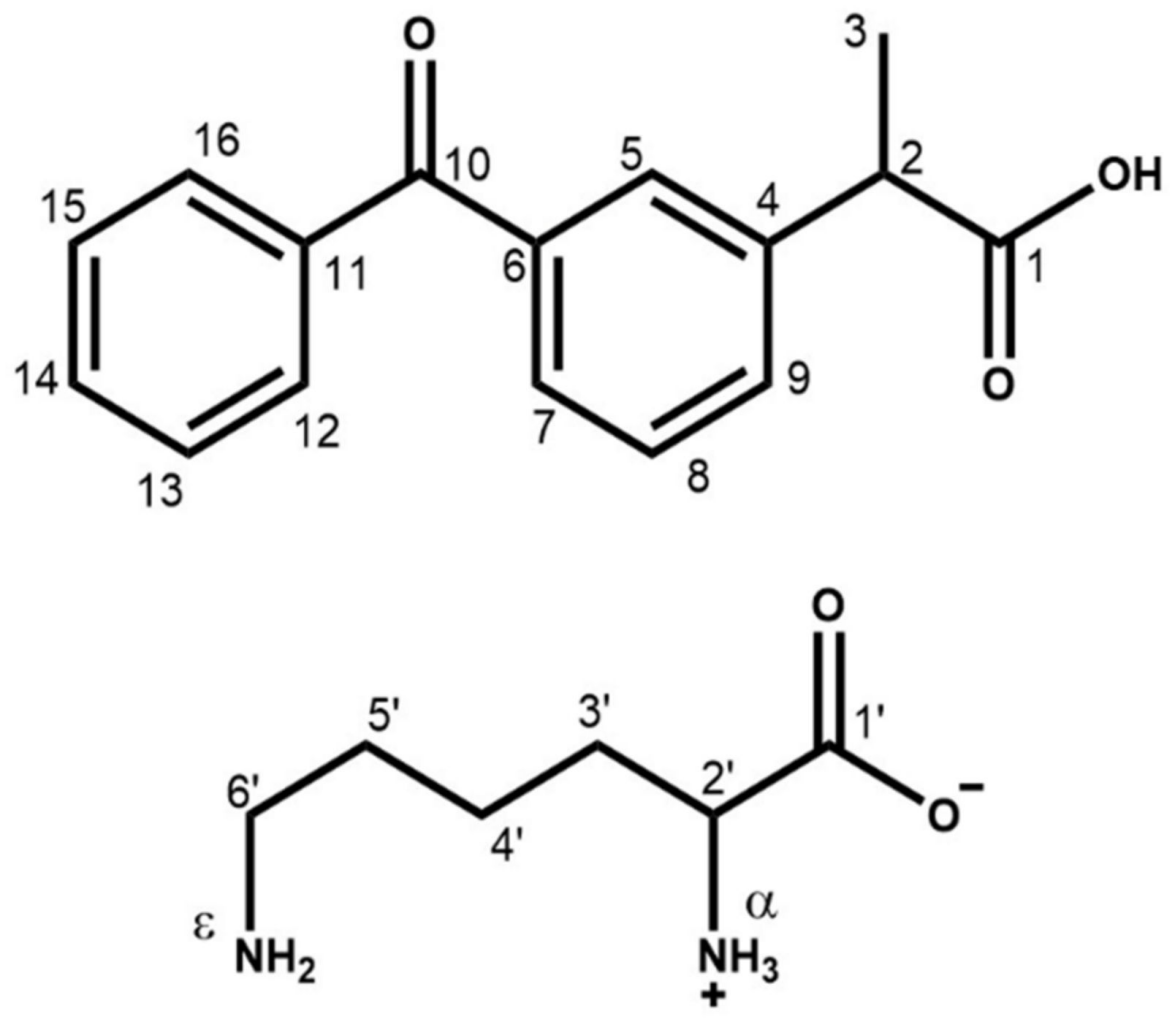
Molecular representation of KET (**top**) and LYS (**bottom**), with C and N atom labeling.

With the aim of studying deeply the solid-state characteristics of ketoprofen–l-lysine salt (KLS) to identify its already known and new crystalline forms and, thus, possibly discover new polymorphic drugs, we thoroughly investigated, for the first time, the existence of polymorphs of the ketoprofen (KET)–lysine (LYS) system (Scheme 1). KET belongs to the group of nonsteroidal anti-inflammatory drugs (NSAIDs), widely used anti-inflammatory, analgesic, and antipyretic compounds that are usually marketed in different forms with sodium, lysine, arginine, and others, to improve the physicochemical and pharmacological profile of the APIs [20,21]. Indeed, salts of KET and LYS, like commercial KLS, have significantly higher solubility compared to KET, allowing for a more rapid absorption of the drug and a subsequent faster onset of the therapeutic effects after oral administration [22].

Here, we report the method of crystallization and characterization of two distinct KET–LYS polymorphic forms, with salt and cocrystal structural characteristics, and we compare their physicochemical properties, their organoleptic characteristics by electronic tongue analysis, and their pharmacokinetics in vivo.

## 2. Results and Discussion

### 2.1. Crystallization Conditions and Identification of KET–LYS Polymorphs

As part of a project aimed at the identification of new polymorphs of the KET–LYS system, we obtained two polymorphic forms of KET–LYS, KET–LYS P1 and KET–LYS P2, through different crystallization techniques (Table S1, Supplementary Materials). We performed more than 230 experiments and analyzed all the collected solids by X-ray powder diffraction (XRPD) analysis and compared them with the starting material. Mechanochemistry methods were ineffective, as grinding and kneading experiments led to the isolation of solids having the same diffraction pattern of the starting material albeit presenting a lower crystallinity degree. Evaporation and slurry experiments in different conditions led to only unstable and amorphous phases. The best results were obtained performing several precipitation experiments by antisolvent addition as described in Section 4. We analyzed the solids obtained using this latter method and deeply characterized them using advanced crystalline solid-state technologies.

### 2.2. Characterization of Synthesized KET–LYS P1 and P2

According to the p*K_a_* rule, the reaction between KET and LYS should result in a salt, as the Δp*K_a_* of these molecules is 5.84 (p*K_a_* (protonated LYS γ-NH_2_) − p*K_a_* (KET COOH) = 10.29 − 4.45). However, since the model suggested by Cruz-Cabeza et al. [10] is validated only for −1 < Δp*K_a_* < 4 and the Δp*K_a_* in this case is higher than 4, the probabilities of obtaining a salt or a cocrystal cannot be quantitatively estimated.

**Figure 1:**
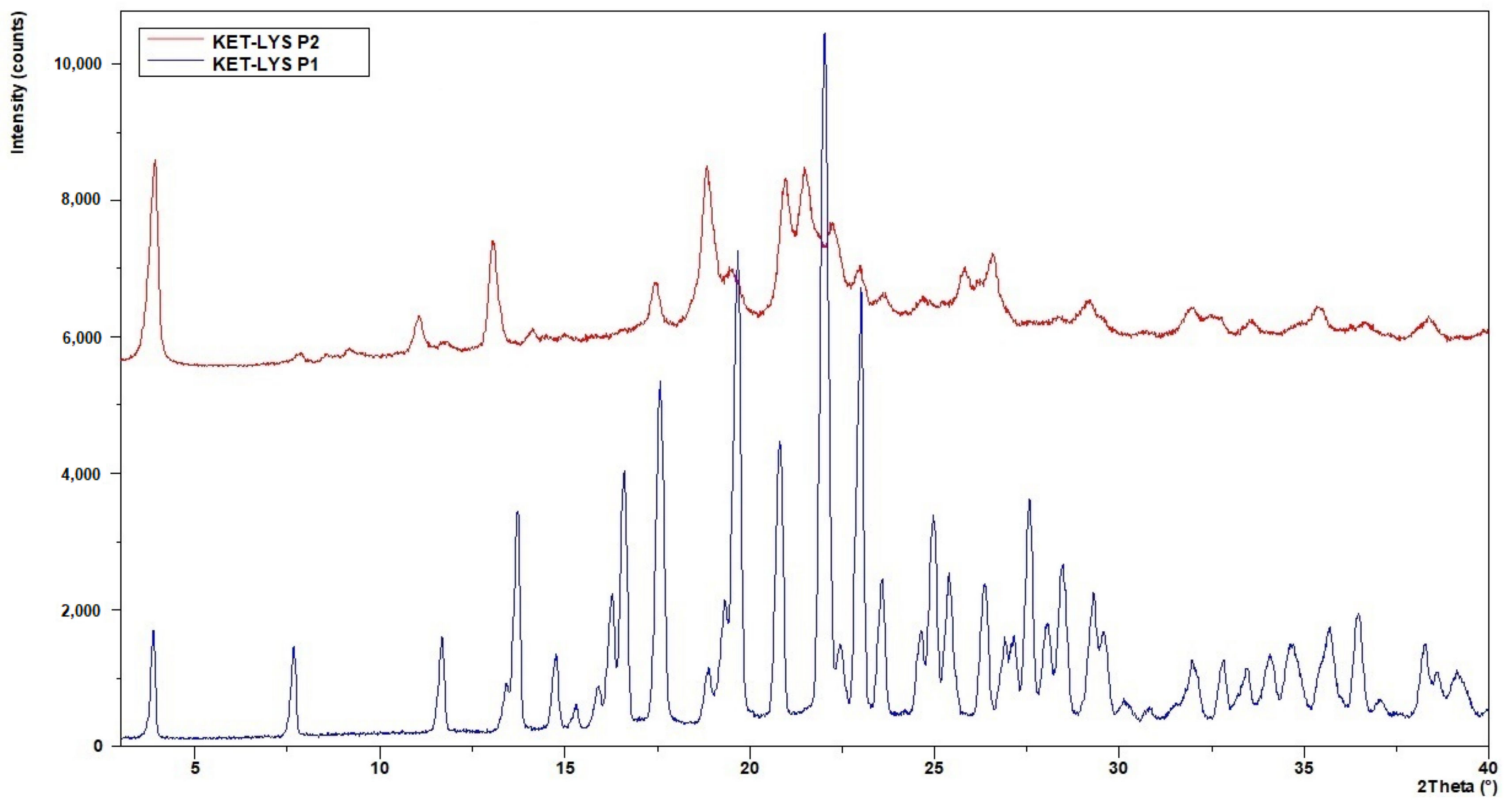
Comparison of XRPD patterns of KET–LYS P1 and KET–LYS P2. The diffraction patterns of KET–LYS P1 and KET–LYS P2 are non-superimposable.

Since crystals of suitable size for single-crystal X-ray diffraction could not be produced, we performed an XRPD analysis to investigate the microstructure and properties of the powders. The non-superimposition of the XRPD diffraction patterns (Figure 1) showed the presence of two different phases with different crystalline degree that were called KET–LYS Polymorph 1 (P1) and KET–LYS Polymorph 2 (P2). From its XRPD pattern, KET–LYS P2 seemed a less crystalline form compared to KET–LYS P1, and we can speculate that this can be due to the faster precipitation of KET–LYS P2 (30 min) compared to that of KET–LYS P1 (3 h).

To define the nature of these two polymorphs, we firstly performed differential scanning calorimetry (DSC). Interestingly, the DSC profile of KET–LYS P1 showed an endothermic event at 170.7 °C (onset 164.1 °C) that was associated with sample melting and degradation (Figure S1, Supplementary Materials), whereas the DSC profile of KET–LYS P2 showed multiple endothermic peaks, with the first one at 110.9 °C (onset 100.5 °C), while, above 120 °C, multiple partially overlapped endothermic peaks were detectable due to degradation steps (Figure S2, Supplementary Materials).

We then performed an FT-IR analysis and observed that the vibration peaks of characteristic functional groups (O–H, N–H, and COOH) of the KET−LYS system significantly differed between the two polymorphs, and this could be attributable to hydrogen bonding and salt formation (Figures S3 and S4, Supplementary Materials). Indeed, in KET–LYS P1, the IR band was centered around 3160 cm^−1^ and could represent the OH stretching in carboxylic acid dimer bands; on the contrary, this band was not detectable in KET–LYS P2, which showed instead a very broad band at 3400–3660 cm^−1^. Lastly, in KET–LYS P2, the strong band at 1.550 cm^−1^ could be due to the KET carboxylate anion group [23].

Starting from this preliminary evidence, we then performed solid-state nuclear magnetic resonance (SSNMR), relying on the accuracy of this technique [24] to undoubtedly determine the ionic or neutral nature of our samples. Both ^13^C and ^15^N chemical shifts are in fact very sensitive to the protonation state of carboxylic and N-containing functional groups [25]. In general, the carboxylic ^13^C chemical shift undergoes a high-frequency shift in the following order: neutral carboxylic groups (COOH) < COOH involved in HB interactions (COOH⋯X) < formation of a dimer through a homosynthon (COOH⋯HOOC) ≈ carboxylate groups (COO^−^) [24]; the ^15^N chemical shift is even more sensitive, as protonation induces shifts that can be as large as 25 ppm toward higher frequencies for aliphatic amines in agreement with the minor contribution of the lone pair to σ^p^_loc_ [24]. Here, 1D (^13^C and ^15^N CPMAS) and 2D (^1^H–^13^C HETCOR) experiments were acquired for KET–LYS P1, and the ^13^C CPMAS spectrum was acquired for KET–LYS P2. For comparison, we also analyzed pure KET, its sodium salt (Na^+^KET^−^), dl-LYS·2HCl, and pure l-LYS, the latter being selected as a reference due to its crystal structure displaying a free ε-NH_2_ group (Figure S5, Supplementary Materials). Table 1 lists ^13^C and ^15^N chemical shifts of KET–LYS P1 and KET–LYS P2 with assignments.

The ^13^C CPMAS spectra of KET–LYS P1 and KET–LYS P2 exhibited consistent shifts in the resonances associated both with KET and with l-LYS carbon atoms (Figure S6, Supplementary Materials). These data are in line with the formation of two novel crystal forms that differed from each other and from the starting materials. The samples were pure, as no residual peaks of the starting materials were present, and both crystal forms contained one independent molecule of KET and one of LYS. The fact that the starting materials were racemic suggests the presence of an inversion center in both crystal forms. Moreover, the average full width at half maximum (FWHM) value for the signals indicated a high and a low degree of crystallinity for KET–LYS P1 (~65 Hz) and KET–LYS P2 (~130 Hz), respectively.

**Figure 2:**
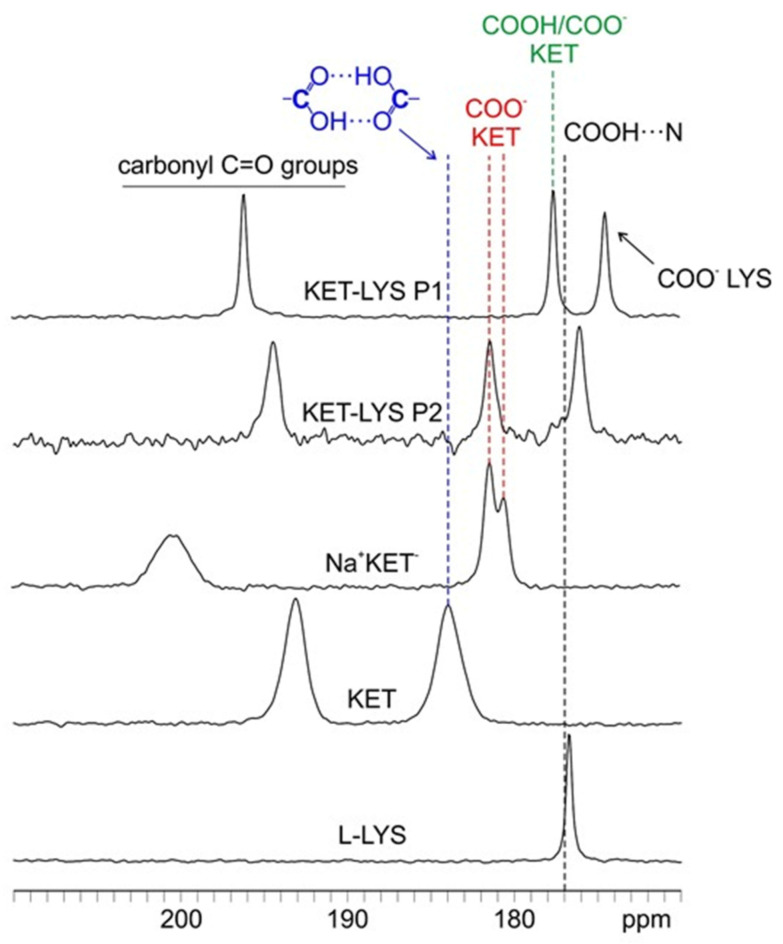
Carboxylic region (170–210 ppm) of the ^13^C-CPMAS spectra of samples KET, l-LYS, Na^+^KET^−^, KET–LYS P1, and KET–LYS P2. The black dashed line at ca. 177 ppm refers to the chemical shift reported in the literature for the protonated carboxylic group of ibuprofen, involved in an HB interaction with a nitrogen atom, in a (ibuprofen)2(4,4′-bipyridyl) cocrystal [27].

In Figure 2, the C=O regions (170–210 ppm) of the ^13^C CPMAS spectra of KET, l-LYS, Na^+^KET^−^, KET–LYS P1, and KET–LYS P2 are reported. Analyzing the ^13^C-CPMAS spectrum of KET, a signal is detectable at 184.0 ppm, a high value that is typical and, thus, indicative of a carboxylic group involved in a homodimeric interaction (as is the case for KET, Figure S7, Supplementary Materials) [26]; on the other hand, its sodium salt Na^+^KET^−^ displays two peaks at 181.4 and 180.5 ppm, which indicate the presence of two independent molecules in the unit cell. Both these chemical shifts are consistent with the unprotonated nature of a carboxylate group (COO^−^). In the case of KET–LYS P1, we observed two peaks in the carboxylic region, at 174.5 and 177.6 ppm.

**Figure 3:**
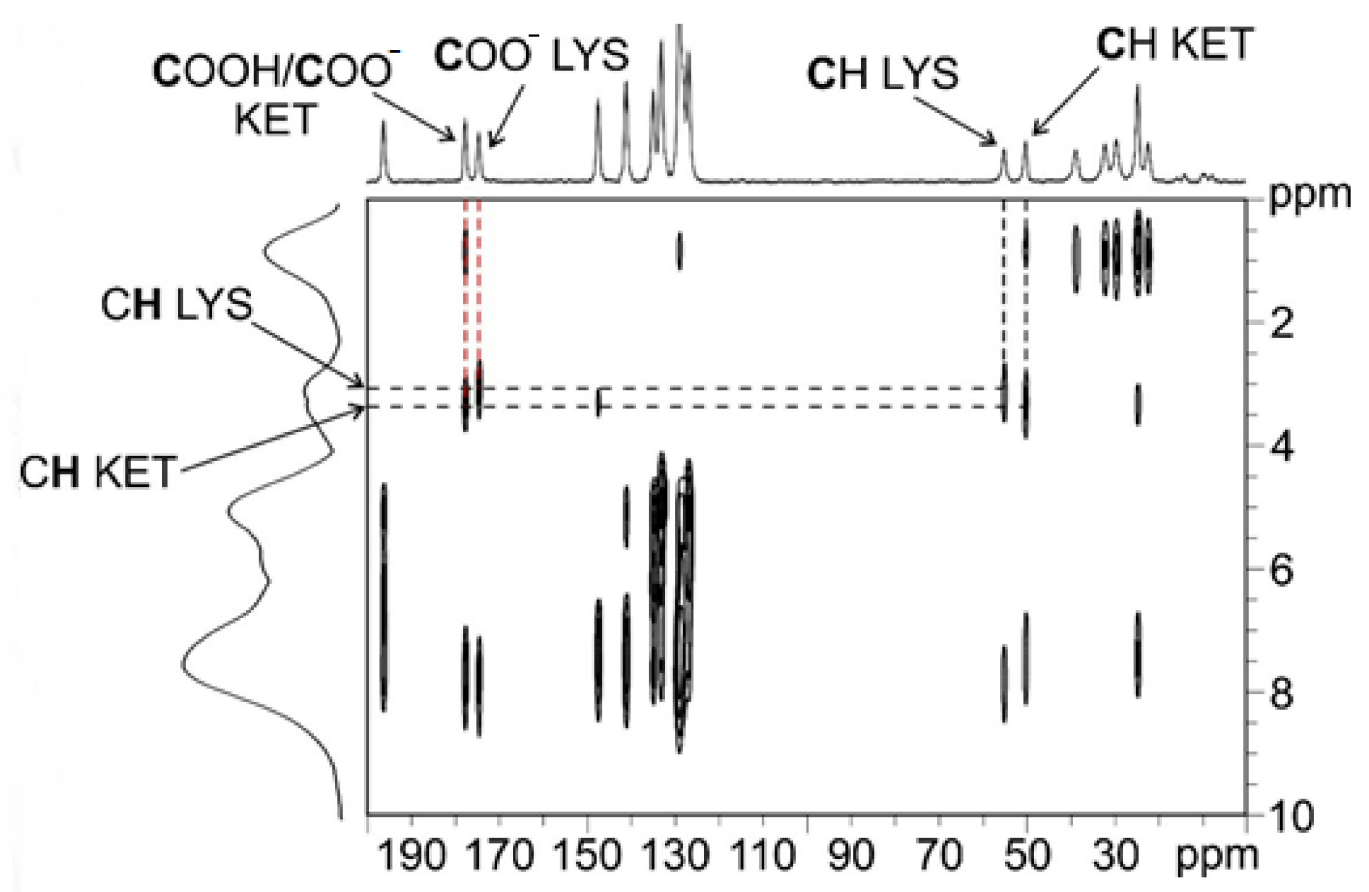
Off-resonance ^1^H–^13^C FSLG HETCOR spectrum (contact time = 7 ms) of KET–LYS P1. Above, ^13^C spectrum; on the left, ^1^H spectrum. Black dashed lines represent significant correlations among covalently bonded protons and C atoms in the crystal structure; red dashed lines represent significant correlations among protons and C atoms spatially close in the crystal structure (see main text). Spinning speed of 12 kHz, room temperature.

To understand which signal refers to the carboxylic group of KET and which refers to that of LYS, we performed a 2D experiment (i.e., ^1^H–^13^C FSLG HETCOR) in two different versions, with on- and off-resonance CP conditions; the first (Figure S8, Supplementary Materials) allowed observing short-range correlations only between covalently bonded C atoms and protons, while the second (Figure 3) also provided signals for long-range interactions between C and H nuclei which were spatially close, within 3–4 Å. The short-range experiment (Figure S8, Supplementary Materials) made it possible to correlate the ^13^C signals of the CH groups of LYS (55.1 ppm) and KET (50.2 ppm) to the corresponding protonic peaks, leading to their assignment at 3.4 and 3.6 ppm, respectively. Being the CH groups closest to the carboxylic moieties in both KET and LYS, we were then able, through the long-range version of the experiment (Figure 3), to identify the carboxylic signal of LYS at 174.5 ppm (correlating with the protonic signal at 3.4 ppm) and that of KET at 177.6 ppm (correlating with the protonic signal at 3.6 ppm). The carboxylic peak of LYS agrees with the typical chemical shift of the COO^−^ in pure LYS (176.7 ppm), which, together with the ^15^N data (see below), confirms the zwitterionic nature of LYS in KET–LYS P1. The KET signal resonates at a frequency which is much lower than that of both the COO^−^ groups of Na^+^KET^−^ (181.4/180.5 ppm) and the homodimeric COOH group of pure KET (184.0 ppm). In particular, the KET signal is very similar to that unambiguously assigned to a neutral carboxylic group involved in an HB, observed at ca. 177 ppm in the (ibuprofen)2(4,4′-bipyridyl) cocrystal [27]. Since the carboxylic environment of ibuprofen is very similar to that of KET, the comparison we made is definitely reliable. Thus, these data suggest that the carboxylic group of KET in KET–LYS P1 is in a protonated state (COOH) and involved in HBs with LYS, making KET–LYS P1 better defined as a cocrystal rather than a salt.

**Figure 4:**
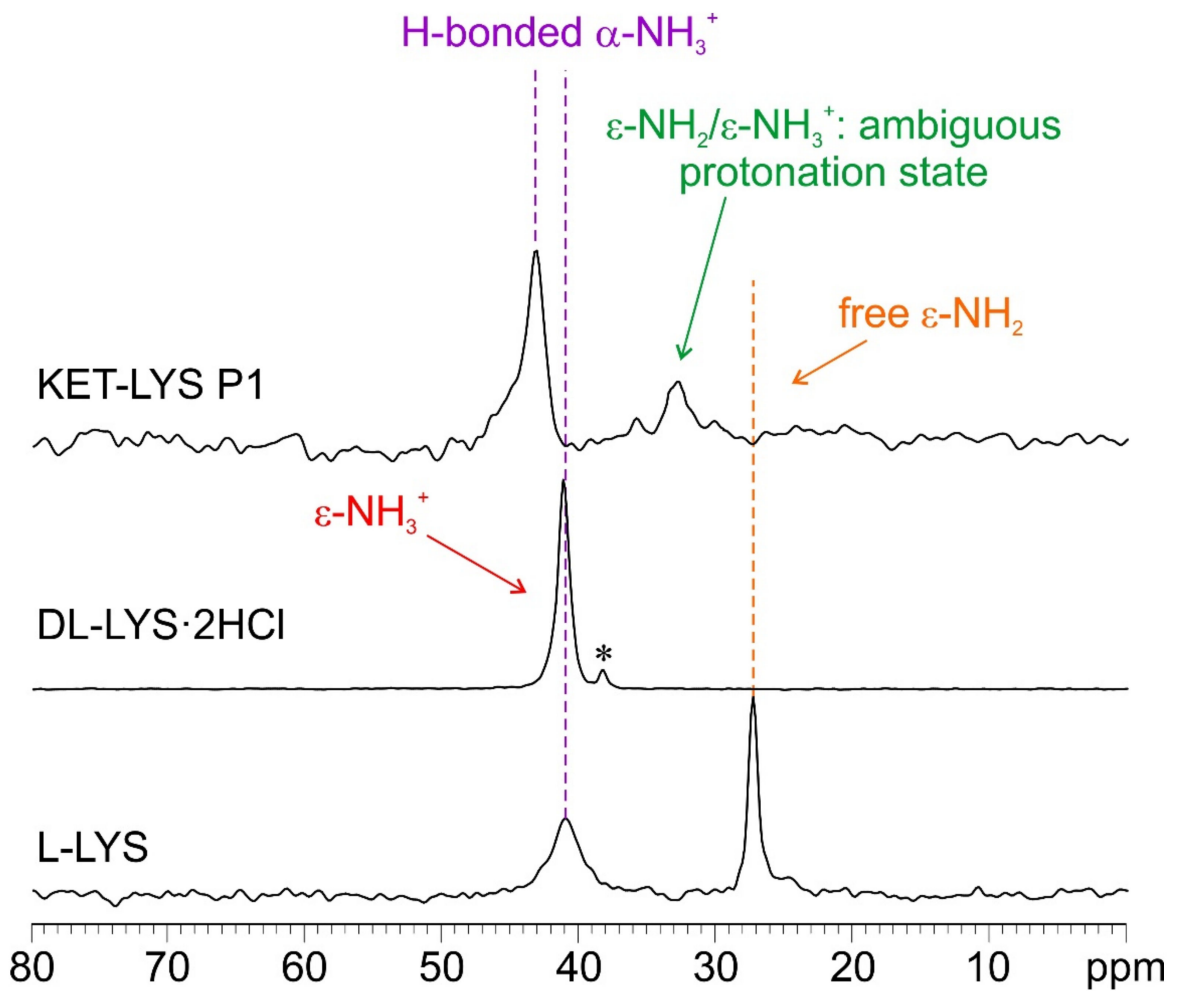
^15^N (40.6 MHz) CPMAS spectra of samples l-LYS, dl-LYS·2HCl, and KET–LYS P1. Acquisition at room temperature at a spinning speed of 9 kHz. The asterisk in the middle spectrum identifies a small signal due to an impurity. The ^15^N CPMAS spectrum of KET–LYS P2 could not be acquired (see Section 4 for further details).

To confirm this assumption, we further investigated the system by ^15^N CPMAS SSNMR analysis, which usually offers the chance to discriminate between deprotonated and protonated N atoms and, thus, in this case, between the NH_2_ and NH_3_^+^ moieties of l-LYS and KET–LYS P1. Figure 4 shows the ^15^N CPMAS NMR spectra of samples l-LYS, dl-LYS·2HCl, and KET–LYS P1. l-LYS itself contains both types of moieties; its ε-NH_2_ group, free from HBs (as observable in its crystal structure, Figure S5, Supplementary Materials; see also [28]), resonates at 27.2 ppm, while its α-NH_3_^+^ falls at 41.0 ppm. The ^15^N-CPMAS spectrum of KET–LYS P1 allowed confirming the protonated nature of the α-NH_3_^+^ of LYS in KET–LYS P1 (43.0 ppm), and it showed that the ε-N signal falls at 32.8 ppm, which is intermediate between the chemical shifts of α-NH_3_^+^ and ε-NH_2_ of pure l-LYS. Although ambiguous, its chemical shift suggests the presence of a neutral NH_2_ group involved in HB interactions, i.e., H_2_N⋯H–X. Indeed, a proper ε-NH_3_^+^ signal falls at 41.3 ppm as observed in the spectrum of dl-LYS·2HCl. Thus, it can be said that this system falls in the salt/cocrystal continuum but points more toward a neutral nature, again supporting the concept that the KET–LYS P1 structure is more referable to a cocrystal rather than to a salt.

From the comparison among the ^13^C CPMAS spectra, it can be observed how, in KET–LYS P2, the signal at 181.5 ppm (referred to KET) is quite close to the signal of pure KET (184.0 ppm) and especially of Na^+^KET^−^ (181.4/180.5 ppm). This suggests that, in KET–LYS P2, the COOH of KET is in a carboxylate form, since dimeric COOH groups and COO^−^ moieties resonate at similar frequencies.

Altogether, these data clearly demonstrate that the crystallization outcome of the reaction between KET and LYS is a case of salt/cocrystal polymorphism. This result is unexpected if we consider the p*K_a_* rule and the fact that the Δp*K_a_* of our system is higher than 4, for which a salt is to be definitely expected. Thus, while the p*K_a_* rule has a statistical and predictive value that is fundamental in the design phase, once the adduct is obtained, the characterization of the protonation state must be always supported by diffraction and/or spectroscopic data. This is because, as also for this system, the influence of the crystalline environment in defining the hydrogen position along an HB is more important than the strength of the acidic and basic sites [11].

Surprisingly, the subsequent analysis of commercial samples of KLS from different manufacturers showed that it is representative of KET–LYS P1 (Figure S9, Supplementary Materials), thus revealing that commercial KLS, which was introduced in the market as a KET salt, should instead be more appropriately defined as a cocrystal. The existence of a stable cocrystal structure of KET–LYS was not predictable on the basis of the typical “p*K_a_* rule”, which has been validated only for a limited Δp*K_a_* range [10]. Thus, these new findings highlight the importance of conducting specific studies to assess the salt/co-crystal polymorphism possibility for species with a Δp*K_a_* outside the −1 < Δp*K_a_* < 4 range.

### 2.3. Intrinsic Dissolution Rates of Cocrystal KET–LYS P1 and Salt KET–LYS P2

**Figure 5:**
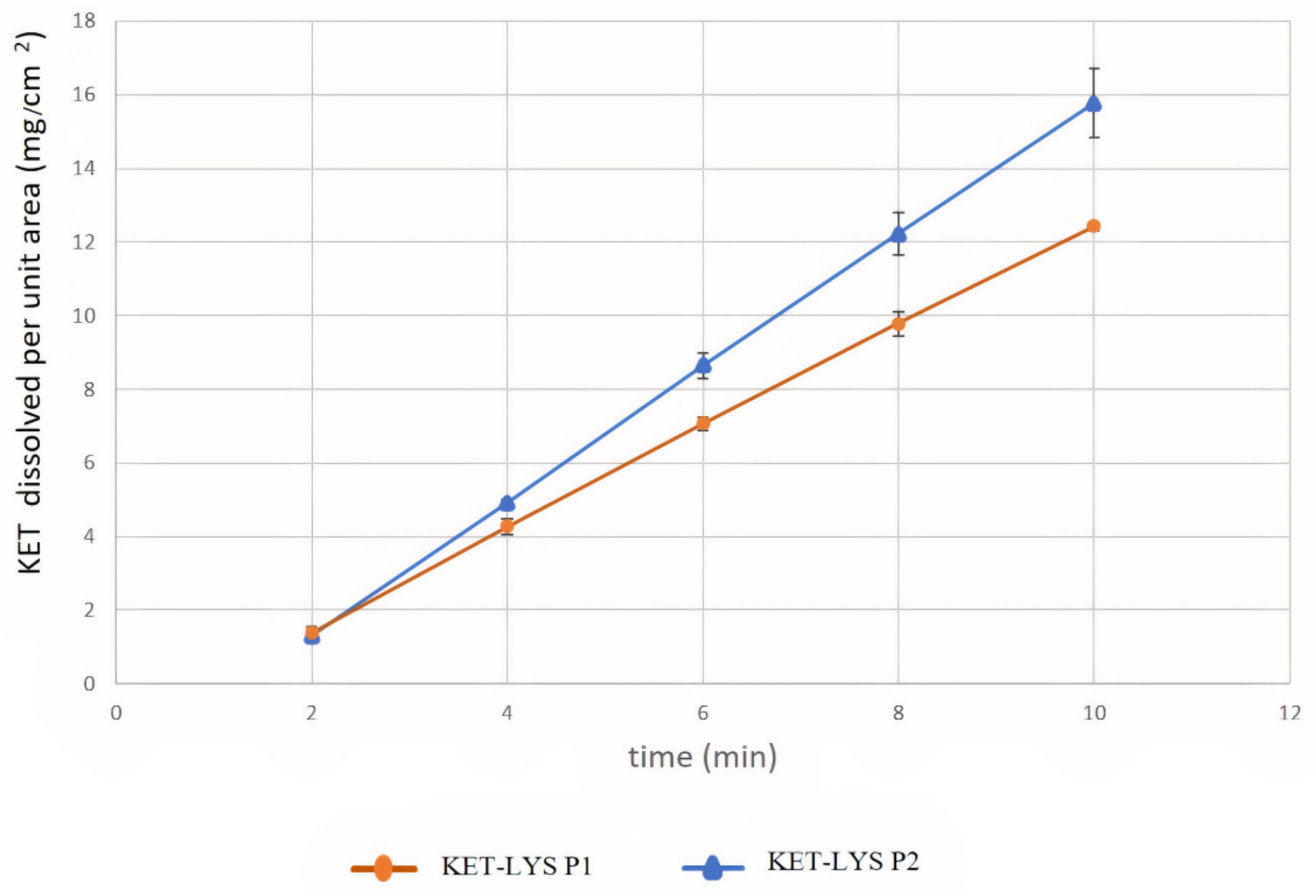
Intrinsic dissolution rates of cocrystal KET–LYS P1 and salt KET–LYS P2. Dissolution profiles of KET from KET–LYS P1 and KET–LYS P2, having constant surface area exposed to the dissolution medium. Three analytical replicates were performed for all the time points. Values are expressed as the mean ± SD.

Having identified two distinct polymorphs of the KET–LYS system, we then analyzed them with regard to some of the intrinsic chemical and physical properties that have to be considered before the development of a pharmaceutical formulation. Among these properties, the dissolution rate of a drug is usually modified by cocrystallization processes [2]. Thus, we investigated whether the cocrystal form of the KET–LYS system (KET–LYS P1) has different intrinsic dissolution rate (IDR) compared to its salt polymorph (KET–LYS P2). The results showed two different IDRs for the two compact forms, having constant surface area exposed to the GSF medium (Figure 5 and Table 2). In particular, the IDR comparative profile (Table 2) between KET–LYS P1 and KET–LYS P2 shows that the release of KET from KET–LYS P2 was significantly faster than from KET–LYS P1 at pH 1.2 (SGF), and KET–LYS P2 seemed to have a higher IDR than KET–LYS P1.

These results further confirm that the outcomes of KET and LYS crystallization are two different structural entities and demonstrate that the two forms are characterized by different IDRs. In vitro dissolution is an important factor in defining drug absorption, distribution, metabolism, and excretion (ADME), and different IDRs among drugs can allow for the development of formulations with different release kinetic profiles [29]. Commercial KLS (representative of KET–LYS P1) has been reported to exhibit fast in vivo absorption and onset of action [22]; thus, the faster dissolution observed for KET–LYS P2 compared to KET–LYS P1 may suggest that the newly synthesized salt form (KET–LYS P2) could be characterized by a faster absorption compared to the commercialized KLS.

### 2.4. Taste and Sensorial Kinetic Analysis of KET–LYS P1 and KET–LYS P2

One of the central challenges of drug manufacturing is to sweeten the unpleasant taste of APIs, which can be bitter, salty, sour, and even metallic or astringent, as it negatively affects the compliance of patients, especially in pediatric and geriatric populations [30]. Technologies have been developed to mask unpleasant drug tastes and odors, such as the use of physical barriers (coating) or the addition of sweeteners and flavoring excipients [31], but modifying the taste of a drug is not an easy, straightforward process, as it strongly depends on the target patient age and geographic location [32] and, of course, on the API itself [33]. Thus, drugs that, at the beginning of their development, have different tastes, will potentially allow for different subsequent coating processes or flavor addition.

As some studies have reported, the taste of a drug is among the chemical properties that cocrystallization can modify [2]. For example, cocrystals of hydrochlorothiazide obtained using sucralose as a coformer had increased dissolution rate and taste masking compared to the API [34], while taste sensing experiments revealed the sweetness of the cocrystal of paracetamol with trimethylglycine due to the presence of the latter in the structure [35], as well as of the cocrystal obtained from theophylline and saccharine [36].

**Figure 6:**
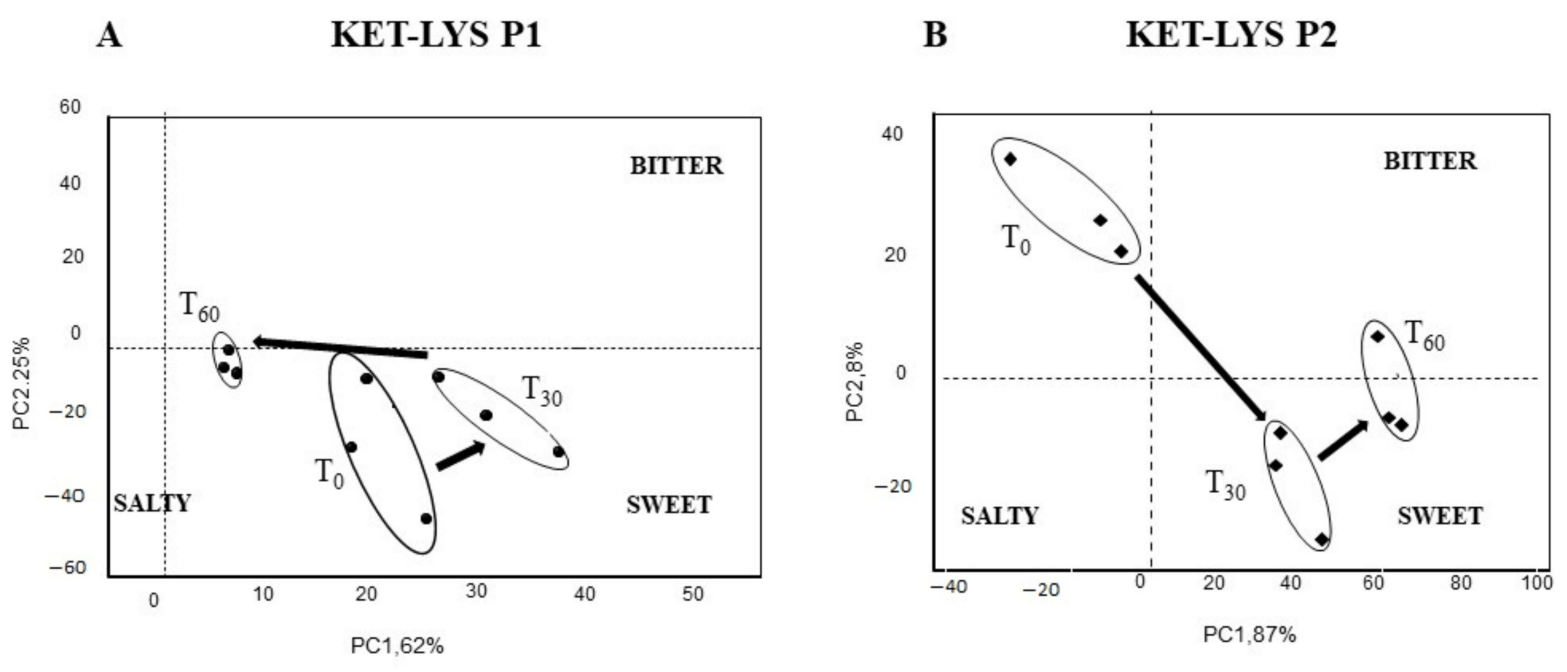
Taste and sensorial kinetic analysis by electronic tongue of cocrystal KET–LYS P1 (**A**) and salt KET–LYS P2 (**B**). PCA score plot of KET–LYS P1 and KET–LYS P2 over the time lapsed after dissolution preparation. T0, just solubilized; T30, 30 min in solution; T60, 60 min in solution. PC1: first principal component; PC2: second principal component. Three analytical replicates were performed for all the time points.

We, thus, investigated and compared the taste and the sensorial kinetics of KET–LYS P1 and KET–LYS P2, to assess whether the substitution of an ionic bond (salt) with a neutral HB (cocrystal), rather than the substitution or the addition of coformers as described in the above examples, can alter these characteristics. To analyze the variation of bitterness and palatability characteristics of KET and LYS pharmaceutical preparations, we used a potentiometric E-tongue system application, a method that was successfully employed in previous researches dedicated to the evaluation of soft cheese salinity [37], water toxicity, and organoleptic potability screening [38,39]. The response of the E-tongue system to the two polymorphs was analyzed by a pattern recognition method called principal component analysis (PCA), to detect the similarities or differences in taste of the sample solutions from time 0 (t0, just solubilized) to 30 min (t30) and after 60 min (t60). The PCA score plot representing the dispersion of E-tongue data obtained for the two polymorphs shows completely different sensorial kinetics between KET–LYS P1 and KET–LYS P2. Firstly, the position on the gustatory map (determined by modeling sweet, bitter, and salty solutions on the PCA score plot) was significantly different at t0 between the cocrystal and the salt forms, as KET–LYS P1 started in the right lower quadrant of the map, while KET–LYS P2 was in the upper part of the left quadrant in an opposite position compared to KET–LYS P1 (Figure 6). These data demonstrate that the cocrystal at t0 is sweeter than the salt and, for this reason, different coating processes and/or flavor addition can be envisioned for the potential subsequent development of the new salt KET–LYS P2 form compared to that usually followed during the manufacturing of the commercialized KLS (KET–LYS P1). Secondly, analysis at t30 and t60 shows that KET–LYS P1 underwent smaller variations in the first 60 min after preparation of the solution compared to KET–LYS P2, as, over time, KET–LYS P1 remained in the same quadrant, while KET–LYS P2 made a significant change in its position on the gustatory map in the first 30 min, moving from the upper part of the left quadrant to the lower right quadrant (Figure 6). This phenomenon could be due to the formation of intimate ion pairs in solution that maintain over time (from t0 to t60) the memory of the different stereochemistry of the original crystalline structures, and this results in a different solvation and shielding of the ion and the counterion of the KET–LYS P1 and KET–LYS P2 systems [40].

### 2.5. Pharmacokinetics In Vivo of KET–LYS P1 and KET–LYS P2

**Figure 7:**
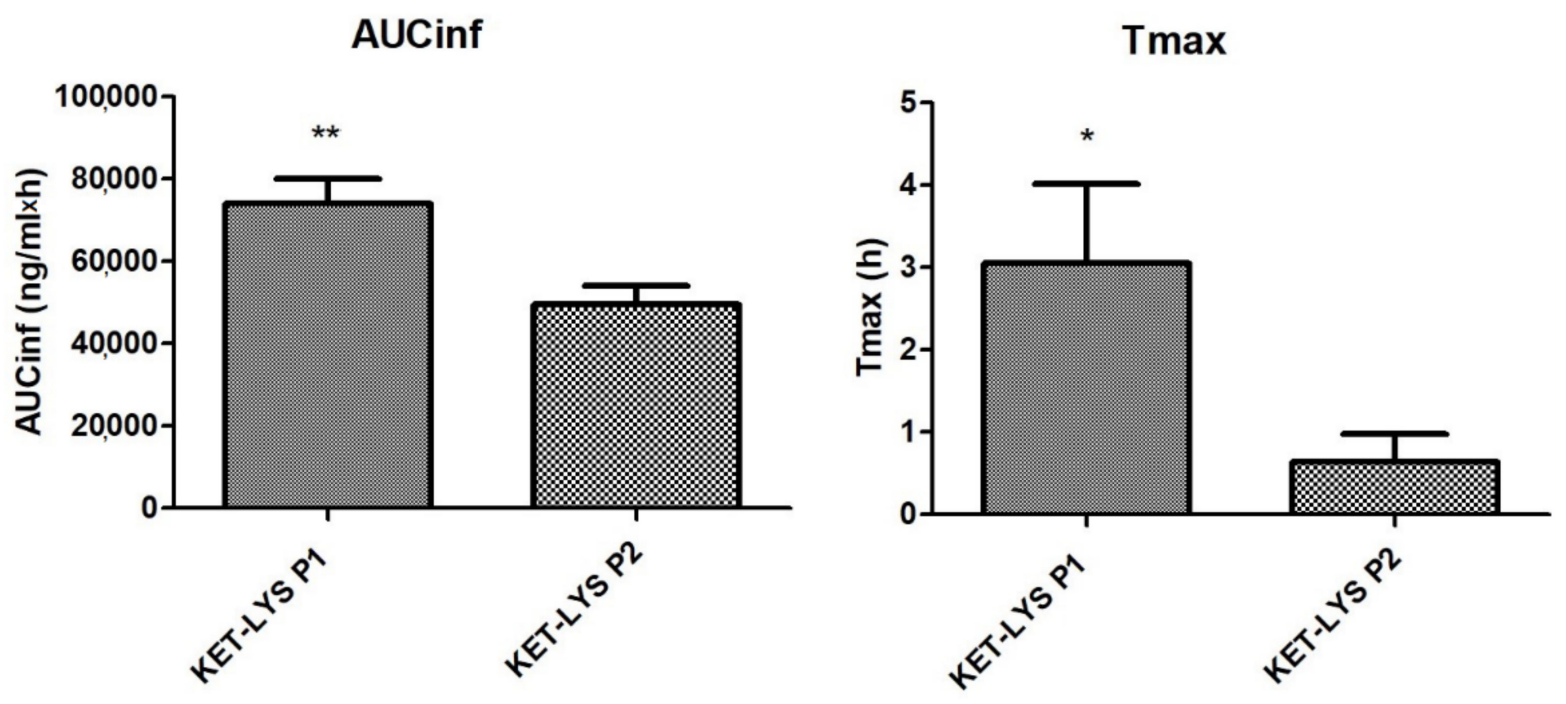
Pharmacokinetics in vivo of cocrystal KET–LYS P1 and salt KET–LYS P2. AUC-inf and T_max_ values of KET–LYS P1 and KET–LYS P2. * *p* < 0.05 and ** *p* < 0.01. Analyses were performed in *n* = 6 rats, and values are expressed as the mean ± SEM.

Having assessed the differences in IDRs between the polymorphic salt and cocrystal forms of KET and LYS, we studied and compared the in vivo pharmacokinetics. We analyzed the major pharmacokinetic parameters after oral administration of the two compounds at the dose of 3.5 mg/kg to not fasted Sprague Dawley male rats. Results showed that the two polymorphs were comparable in terms of C_max_, T_1/2_, and mean residence time (MRT); however, notably, the dose-normalized area under the curve to infinity (AUC-inf) and T_max_ values of KET–LYS P1 were significantly higher than those of KET–LYS P2 (74,076 ± 5931 ng/mL·h vs. 50, 434 ± 4439 ng/mL·h, *p* < 0.01, for AUC-inf, and 3.1 ± 0.97 h vs. 0.64 ± 0.34 h, *p* < 0.05 for T_max_, for P1 and P2, respectively, *n* = 6, mean ± SEM) (Figure 7). After single oral administration, AUC-inf measures the absorbed drug amount and is calculated from administration time to infinity (inf), whereas the peak time (T_max_) depends on the rate of drug absorption. Thus, the lower AUC-inf of KET–LYS P2 compared to that of commercial KET–LYS P1 could probably mean a lower bioavailability of KET–LYS P2, in addition to a lower T_max_ that indicates a faster absorption of this latter compared to commercial KLS. These data agree with IDR data and show the potential advantages of KET–LYS P2 for a further improvement of fast-acting formulations for the treatment of acute inflammation conditions.

While it can be expected that cocrystals and salts with different coformers have different physicochemical and pharmacokinetic characteristics, finding significant differences in the IDR, taste, and pharmacokinetics of a salt and its cocrystal polymorph surely opens new perspectives. Altogether, these differences clearly highlight the actual impact that (in this case) the substitution of one ionic bond (salt) with a neutral HB (cocrystal) can have on drug clinical performance, to the extent that different formulations and different coating processes or flavor addition can be suggested for the two polymorphs. KLS is a widely used NSAID that is already known, as also confirmed by our data on KET–LYS P1 T_max_, for its rapid absorption and subsequent therapeutic action [22], which make it indicated for the treatment of acute inflammatory and painful conditions. From our data, we can hypothesize that new polymorphic form KET–LYS P2 could further emphasize the property of KLS for the treatment of acute inflammatory and painful conditions.

## 3. Materials and Methods

### 3.1. Crystallization Conditions Screened to Identify Potential Polymorphs

To deeply investigate the solid-state characteristics of KLS, we conducted a polymorph screening study to identify all the crystalline forms that this API can adopt. To this end, several crystallization conditions were tested on the basis of the solubility properties of the compounds (Table S1, Supplementary Materials). Experiments were performed by (i) dry grinding, in which solid forms of both coformers were combined for manual/mechanical grinding for a fixed period, (ii) kneading (liquid assisted grinding), in which solid forms of both coformers were combined in the presence of a very small amount of solvent for manual/mechanical grinding for a fixed period, (iii) slurry, in which solid forms of both coformers were added to a solvent/solvent mixture for a fixed period of equilibration with the solid remaining in excess for the duration of the experiment, (iv) evaporation at low/room/high temperature (based on boiling point), in which the solvent was removed from an undersaturated solution of both coformers via evaporation at various temperatures, and (v) precipitation by antisolvent addition, in which cocrystallization directly resulted after the addition of an antisolvent to a solution of both coformers.

For all the compounds, various stoichiometric ratios were investigated in the range of 0.5 to 2 equivalents with respect to the amount of KET or LYS. The slurry experiment with saturated solutions in various solvents was performed when possible.

### 3.2. General Procedure for the Preparation of KET-LYS Polymorph 1 (P1)

First, 50 g of (*RS*)-KET was added to 350 mL of ethanol, and the mixture was stirred at RT until complete solubilization. Then, 29 g of dl-LYS, 50% *w/w* in water (1:1 ratio), was added, and the solution was stirred at RT until the first precipitation occurred. The mixture was left under these conditions for 3 h and then cooled to 5 °C. After 5 h, the product was recovered by filtration, washed with 60 mL of ethanol, and dried under reduced pressure at 40 °C. The final KET–LYS P1 was obtained as a white powder (69 g, yield of 88%).

### 3.3. General Procedure for the Preparation of KET–LYS Polymorph 2 (P2)

First, 1.2 g of (*RS*)-KET and 0.69 g of dl-LYS (1:1 ratio) were suspended in 20 mL of methanol and left under stirring at 40 °C for 1 h. The suspension was then filtered (0.45 μm filter) directly in a Mettler Toledo Easymax 102 reactor. The solution was left under stirring for 5 min in the reactor; then, 100 mL of ethyl acetate was added, and the solution was cooled to −5 °C without solid formation. Ethyl acetate (20 mL) was added through a pipette in two aliquots (10 mL and 10 mL) to trigger nucleation. The system was left under stirring until the suspension became “milky”. An additional 30 min of stirring was applied. The precipitate was then filtered, and the collected sample was stored in a sealed vial at room temperature. The final KET–LYS P2 was obtained as a white powder (1.3 g, yield of 69%).

### 3.4. X-ray Powder Diffraction (XRPD)

XRPD experiments were performed on a powder X-ray diffractometer (Rigaku MiniFlex600, Applied Rigaku Technologies, Inc., Austin, TX, USA) using Cu Kα radiation (1.540598 Å). Samples were scanned with a step size of 0.01° (2***θ***) and speed of 10.0°/min (2***θ***) from 3° to 40° 2θ. The tube voltage and amperage were 40 kV and 15 mA, respectively.

### 3.5. Thermal Analyses

The analyses were carried out using the Mettler Toledo TGA/DSC1. Samples were weighed in an aluminum pan hermetically sealed with an aluminum pierced cover. The analyses were performed heating the sample from 25 °C to 320 °C at 10 °C/min.

### 3.6. Fourier-Transform Infrared Spectroscopy (FT-IR)

The analysis was carried out using a Thermo Nicolet iS50 ATR module spectrometer equipped with a Smart Performer Diamond, DTGS KBr detector, IR source, and KBr beam splitter.

### 3.7. Solid-State NMR Characterization

Solid-state NMR (SSNMR) spectra of KET, LYS, Na^+^KET^−^, and KET–LYS P1 were acquired with a Bruker Avance II 400 Ultra Shield instrument, operating at 400.23, 100.63, and 40.56 MHz, respectively, for ^1^H, ^13^C, and ^15^N nuclei.

Powder samples were packed into cylindrical zirconia rotors with a 4 mm o.d. and an 80 μL volume. A certain amount of sample was collected from each batch and used without further preparation to fill the rotor. **^13^**C CPMAS spectra were acquired at a spinning speed of 12 kHz, using a ramp cross-polarization pulse sequence with a 90° ^1^H pulse of 3.60 μs, a contact time of 3 ms, optimized recycle delays between 1.5 and 3.5 s, and a number of scans in the range 430–640, depending on the sample. **^15^**N CPMAS spectra were acquired at a spinning speed of 9 kHz using a ramp cross-polarization pulse sequence with a 90° ^1^H pulse of 3.60 μs, a contact time between 1 and 4 ms, optimized recycle delays between 1.1 and 3.4 s, and a number of scans in the range 14,330–22,770, depending on the sample. For every spectrum, a two-pulse phase modulation (TPPM) decoupling scheme was used, with a radiofrequency field of 69.4 kHz. The ^13^C chemical shift scale was calibrated through the methylenic signal of external standard glycine (at 43.7 ppm). The ^15^N chemical shift scale was calibrated through the signal of external standard glycine (at 33.4 ppm with reference to NH**_3_**).

The 2D ^1^H−^13^C on- and off-resonance (short and long range, respectively) HETCOR spectra were measured with contact times of 0.1 and 7 ms, respectively, and FSLG t1 decoupling and TPPM t2 decoupling (rf fields of 82 kHz). Accordingly, 288 and 384 scans were averaged for 88 and 128 increments, respectively, with 3.4 s of relaxation delay. The indirect ^1^H chemical shift scale in the HETCOR spectra was experimentally corrected by a scaling factor of 1/√3 because the ^1^H chemical-shift dispersion is scaled by a factor of 1/√3 during FSLG decoupling.

The ^13^ CPMAS spectrum of KET–LYS P2 was acquired with a Jeol ECZR 600 instrument, operating at 600.17 and 150.91 MHz, respectively, for ^1^H and ^13^C nuclei. The powder sample was packed into a cylindrical zirconia rotor with a 3.2 mm o.d. and a 60 μL volume. A certain amount of sample was collected from the batch and used without further preparations to fill the rotor. The ^13^C CPMAS spectrum was acquired at 273 K, at a spinning speed of 20 kHz, using a ramp cross-polarization pulse sequence with a 90° ^1^H pulse of 2.19 μs, and a contact time of 3.5 ms. An optimized recycle delay of 6 s was used, for a number of scans of 240. A two-pulse phase modulation (TPPM) decoupling scheme was used, with a radiofrequency field of 108.5 kHz. The ^13^C chemical shift scale was calibrated through the methylenic signal of external standard glycine (at 43.7 ppm).

The well-known ^15^N-NMR low sensitivity together with the poorly crystalline nature of KET–LYS P2 prevented a proper parameter optimization of the CPMAS experiment. Several attempts were made for acquiring the ^15^N CPMAS spectrum of KET–LYS P2 by changing several parameters (contact time, number of scans, and Hartmann–Hahn conditions), without succeeding.

### 3.8. Intrinsic Dissolution Rate

KET–LYS P1 and KET–LYS P2 were tested for intrinsic dissolution rate (IDR). The IDR experimental method was performed using a 150 mg powder sample; this was compacted by means of a hydraulic press in a round Ø = 11 mm matrix, under approximately 2 tons of force for 3 min. The obtained compacts were maintained inside the matrix and tested in a USP42 Apparatus 2 (Distek Dissolution System 2100B, Distek, Inc., NJ, USA) under the following conditions: 500 mL of gastric simulated fluid (GSF) without pepsin, 37 °C, and 30 rpm paddle rotation speed. The amount of solid dissolved at each time point was determined spectrophotometrically at 260 nm. The test was performed in three replicates. Statistical analysis of the data was performed by Microsoft Excel.

### 3.9. Multisensory Analysis

The composition and properties of tested samples (KET–LYS P1 and KET–LYS P2) are listed in Table S2 (Supplementary Materials). The response of E-tongue system was tested in model solutions of salty, bitter, and sweet taste for which aqueous solutions of 0.001 mol/L NaCl, 0.001 mol/L MgCl_2_, and 0.06 mol/L of fructose were used, respectively. Millipore-grade water was used for aqueous solution preparation. All the other chemicals were of analytical grade and used without further purification.

Membrane components, high-molecular-weight poly(vinyl chloride) (PVC), bis(2-ethylhexyl) sebacate (DEHS) plasticizer, tridodecylmethyl ammonium chloride (TDMACl), potassium tetrakis-(4-chlorophenyl)borate (TpClPBK) lipophilic additives, and nonactine ionophore were purchased from Sigma-Aldrich (Rome, Italy). Tetrahydrofuran (THF) used for PVC membrane preparation was obtained from Carlo Erba Reagents (Rome, Italy) and distilled prior to use. 5,10,15,20-Tetraphenylporphyrin manganese(III) chloride ionophore (Mn(TPP)Cl) was synthesized in our laboratories and fully characterized according to the literature procedure [41]. Millipore-grade water was used for aqueous solution preparation. All the other chemicals were of analytical grade and used without further purification.

The potentiometric E-tongue system was composed of eight sensors with three different types of sensing membranes: PVC-based solvent polymeric membranes doped with Mn(TPP)Cl (sensor A1) and nonactin (sensor C1) ionophores, chalcogenide glass membranes doped with different metal salts (G2-Cu, G7-Tl, G8-Ag, G10-Cd, G11-Pb), and sensor A7 with polycrystalline LaF_3_ membrane. The PVC-based solvent polymeric membranes were prepared according to the previously reported procedures [42,43]. For this, all the membrane components (PVC 30–33 wt.%, plasticizer 60–66 wt.%, ion-exchanger 0.1–10 wt.%, and ionophore 1 wt.%) were dissolved in THF. The membrane cocktails were then cast in a 24 mm i.d. glass ring on a glass slide, and the solvent was evaporated overnight. The polymeric membrane discs of 8 mm in diameter were cut out from the parent membrane and fixed with 10 wt.% PVC in cyclohexanone glue onto hollow PVC tubes that served as electrode bodies, filled with 0.01 mol/L solutions of NaCl and NH_4_C1 for sensor A1 and sensor C1, respectively. The sensor A1 and C1 membrane potential was registered against the internal, homemade Ag/AgCl reference electrode.

Chalcogenide glass sensors and sensor A7 with a polycrystalline LaF_3_ membrane had solid Cu-wire/Ag-paste solid contacts and were purchased from Sensor Systems (St. Petersburg, Russia). The potentials of E-tongue system sensors were measured versus a saturated calomel reference electrode (SCE, AMEL, Milan, Italy), in a standard two-electrode configuration cell. Potentiometric measurements were performed with a LiquiLab (ECOSENS srl, Rome, Italy) high-impedance analog-to-digital potentiometer. Prior to measurements, the sensors were soaked in 0.01 mol/L NaCl aqueous solution for at least 24 h.

The quantity of samples KET–LYS P1 and KET–LYS P2 corresponded to the standard dosage of administration of commercial KLS (40 mg). This amount of sample was dissolved in 20 mL of distilled water.

All analyses were performed in analytical triplicate for all the above time conditions. The pH value and electrical conductivity of the t24 h and t48 h solutions were also measured. The samples were stored in closed containers at room temperature (+22 °C) during the study period.

### 3.10. Pharmacokinetics

For the pharmacokinetics studies, Sprague Dawley male rats (body weights 250–275 g at the time of the treatment) were used. The animals were originally supplied by Harlan, Italy. The animals were acclimatized to local housing conditions for approximately 5 days.

The animals were housed in a single, exclusive room, air-conditioned to provide a minimum of 15 air changes/h. The environmental controls were set to maintain temperature within 22 °C and relative humidity within the range 50–60% with an approximate 12 h light and 12 h dark cycle that was controlled automatically. Food (Mucedola Standard GLP diet) and water were available ad libitum throughout the study. All animals were weighed on the day of each treatment. Clinical signs were monitored at regular intervals throughout the study in order to assess any reaction to treatment. Each animal was uniquely identified with a colored spray on the back before the experiment.

The compounds were administered at a dose of 3.5 mg/kg as KET free acid in gelatin capsules (size 9, Torpac^®^). To perform this, capsules were individually filled and weighed up with the substances and then filled with rice starch up to about 20 mg, weighed again, and closed. After administration, blood (approximately 60–80 µL) was sampled from tail vein at the following timepoints: 15 min, 30 min, 1 h, 2 h, 4 h, 6 h, 8 h, 24 h, and 48 h. Blood samples were collected in heparinized Eppendorf tubes (Heparin Vister 5000 UI/mL), gently mixed, and placed immediately on ice; then, Eppendorf tubes were centrifuged (3500× g, at 4 °C for 15 min), and the resulted plasma was collected and transferred to uniquely labeled Eppendorf tubes, before being frozen at −20°C till the HPLC–MS analysis.

At the end of the study animals were sacrificed by exsanguination under deep isoflurane anesthesia.

The experiment was carried on in agreement with the Italian Law D. L.vo 4 marzo 2014, no. 26.

### 3.11. Statistics

For IDR experiments, the statistical analysis of the data was performed by Microsoft Excel (Microsoft Excel for Office 365 MSO (16.0.11929.20836)). For pharmacokinetics experiments, data were expressed as the mean ± standard error of the mean (SEM). Multiple statistical comparisons between groups were performed by two-tailed unpaired Student’s *t*-test. All data collected followed good approximation to a normal distribution, being included in the 95% confidence interval of the mean; this generally allowed for the clear identification of outliers, if any, and for the application of the statistical analyses described above. No outliers were found during the present study. Missing data in the results were then related only to overt technical issues during the experimental procedures, which led to the exclusion of those specific samples from the analysis.

## 4. Conclusions

In this study, we synthesized and characterized, for the first time, two different polymorphic forms of KLS, a cocrystal, KET–LYS P1, and a salt, KET–LYS P2. Through advanced and sophisticated analyses, we were able to undoubtedly define the structural characteristics of the two polymorphs and compare their physicochemical characteristics, in terms of dissolution rate and taste, and pharmacokinetic profiles in vivo. What emerged is that, from a structural point of view, the commercial KLS, which was introduced in the market as a KET salt, should instead be more appropriately defined as a cocrystal (KET–LYS P1), and that the newly identified salt KET–LYS P2 has significantly different chemical and pharmacokinetic characteristics compared to KET–LYS P1. The faster dissolution rate and reduced T_max_ in vivo of KET–LYS P2 suggest potential advantages of this new form for fast-acting formulations of the drug. Nevertheless, it should be considered that this new polymorph may exhibit a more bitter taste and a lower AUC compared to the commercially available KLS form, and this should prompt the design of specific formulation studies to hypothesize different potential formulations (faster release) and coating or flavoring processes, according to the specific characteristics of the starting API material.

The present study advances our knowledge regarding the solid-state characteristics of KLS and gives a comprehensive view of its polymorphic diversity; while doing this, it led to the discovery of a new polymorph of KLS, KET–LYS P2, thus opening the way for the development of a new potential KET–LYS polymorph drug, the pharmacological and pharmacokinetic characteristics of which will be further investigated to define the appropriate formulation and the conditions that would most benefit from the treatment with this drug.

## Figures and Tables

**Table 1:** ^13^C and ^15^N SSNMR chemical shift assignments for KET–LYS P1 and KET–LYS P2. Please refer to Scheme 1 for atom labeling. The ^15^N CPMAS peak assignment for KET–LYS P2 is not reported as the spectrum could not be acquired (see Section 3 for further details).

KET–LYS P1		KET–LYS P2	
**^13^C**			
**^13^C δ (ppm)**	**C atom**	**^13^C δ (ppm)**	**C atom**
196.1	10	194.5	10
177.6	1	181.5	1
174.5	1′	176.1	1′
147.4	6	145.1	6
141.0	11	139.7	11
134.8	4	138.1	4
133.0	Aromatic CH	133.4	Aromatic CH
128.8	Aromatic CH	131.8	Aromatic CH
128.3	Aromatic CH	129.8	Aromatic CH
128.0	Aromatic CH	128.8	Aromatic CH
126.8	Aromatic CH	127.7	Aromatic CH
55.1	2′	53.2	2′
50.2	2	47.4	2
38.8	6′	34.5	6′
32.2	5′	25.9	5′ + 3′
29.6	3′	21.2	3
24.7	3	16.6	4′
22.3	4′	/	/
**^15^N**			
**^15^N δ (ppm)**	**N atom**		
43.0	α		
32.8	ε		

**Table 2:** IDR (slope) mg/(cm^2^ **×** min) of cocrystal KET–LYS P1 and salt KET–LYS P2.

Compound	IDR (Slope) mg/(cm^2^ × min)	Confidence Interval (95%)	
**Cocrystal KET–LYS P1**	1.453	1.411	1.495
**Salt** **KET–LYS P2**	1.907	1.813	2.002

## Data Availability

The data presented in this study are available on request from the corresponding author.

## Supplementary Materials

The following are available online at https://www.mdpi.com/article/10.3390/ph14060555/s1: Table S1. Tested samples and model taste solutions for E-tongue analysis; Figure S1. DSC and TGA of KET–LYS P1; Figure S2. DSC and TGA of KET–LYS P2; Figure S3. FT-IR spectrum of KET–LYS P1; Figure S4. FT-IR spectrum of KET–LYS P2; Figure S5. Crystal structure of l-lysine; Figure S6. ^13^C CPMAS spectra of samples KET, l-LYS, Na^+^KET^−^, dl-LYS·2HCl, KET–LYS P1, and KET–LYS P2; Figure S7. Crystal structure of (*RS*)-KET displaying the typical carboxylic homodimeric synthon; Figure S8. On-resonance ^1^H–^13^C FSLG HETCOR spectrum (contact time = 0.1 ms) of KET–LYS P1; Figure S9. XRPD pattern comparison between KET–LYS P1 and commercial KLS samples.

## Patents

The composition, process of production, and uses of cocrystal polymorph of KLS (KET–LYS P1) are described in Patent number WO2020126088A1.
