# Supplementary material for: Unexpected Salt/Cocrystal Polymorphism of the Ketoprofen–Lysine System: Discovery of a New Ketoprofen–l-Lysine Salt Polymorph with Different Physicochemical and Pharmacokinetic Properties
Source: Pharmaceuticals (Basel). 2021 Jun 10;14(6):555. doi: 10.3390/ph14060555 (PMC8230491; doi:10.3390/ph14060555)
Supplement: Supplementary file 1 [file pharmaceuticals-14-00555-s001.zip › pharmaceuticals-1240671-supplementary.pdf]

Table S1. Representative examples of all the crystallization techniques.

Entry	Stochiometric ratio (KET:LYS)	Experiment Type	Solvent	XRPD	Notes
1	1:1	GR	-	KET + LYS	Amorphous
2	1:2	GR	-	KET + LYS	Amorphous
3	2:1	GR	-	KET + LYS	Amorphous
4	1:1	KN	Ethanol	KET + LYS	Low crystallinity degree
5	1:1	KN	Methanol	KET + LYS	Low crystallinity degree
6	1:1	KN	2-Propanol	KET + LYS	Amorphous
7	1:1	KN	Acetonitrile	KET + LYS	Amorphous
8	1:1	EvRT	Acetonitrile	-	LYS not soluble
9	1:1	EvHT	Anisole	Sticky solid	
10	1:1	EvHT	DMF	KET	
11	1:1	EvHT	DMSO	KET	
12	1:1	EvRT	Dichloromethane	Sticky solid	
13	1:1	EvRT	Chloroform	-	LYS not soluble
14	1:1	EvRT	1,2-Dimethoxy Ethane	-	LYS not soluble
15	1:1	EvRT	Diethyl Carbonate	-	LYS not soluble
16	1:1	EvRT	Isopropyl Acetate	-	LYS not soluble
17	1:1	EvRT	Methyl Ethyl Ketone	-	LYS not soluble
18	1:1	SLRT	Acetonitrile	Sticky solid	
19	1:1	SLRT	Ethanol	Sticky solid	
20	1:1	SLRT	Methanol	Amorphous	
21	1:1	SLRT	DMF	Amorphous	
22	1:1	SLRT	DMSO	Amorphous	
23	1:1	SLRT	Dichloromethane	KET + LYS	
24	1:1	PAD	1-Butanol	KET + LYS	
25	1:1	PAD	1-Pentanol	KET + LYS	
26	1:1	PAD	1-Propanol	Sticky solid	
27	1:1	PAD	2-Butanol	Sticky solid	
28	1:1	PAD	2-Methoxy Ethanol	KET + LYS	
29	1:1	PAD	2-Propanol	Sticky solid	
30	1:1	PAD	Acetonitrile	KET-LYS P1	Low crystallinity degree
31	1:1	PAD	Acetone	KET-LYS P1	Low yield
32	1:1	PAD	1,4-Dioxane	KET-LYS P1 + LYS	
33	1:1	PAD	N,N-Dimethylacetamide	no precipitation	
34	1:1	PAD	N,N-Dimethylformamide	no precipitation	

35	1:1	PAD	Dimethylsulfoxide	no precipitation	
36	1:1	PAD	Ethanol	KET-LYS P1	Selected procedure for P1
37	1:1	PAD	Methanol	KET-LYS P1	
38	1:1	PAD	Tetrahydrofuran	no precipitation	
39	1:1	PAD	1-Butanol	KET-LYS	Low crystallinity degree
40	1:1	CRY	1-Pentanol	KET-LYS P1 + LYS	
41	1:1	CRY	1-Propanol	KET-LYS P1 + LYS	
42	1:1	CRY	2-Butanol	KET-LYS P1 + LYS	
43	1:1	CRY	2-Methoxy Ethanol	Sticky solid	
44	1:1	CRY	2-Propanol	Amorphous	
45	1:1	CRY	Acetonitrile	Sticky solid	
46	2:1	CRY	Acetone	Sticky solid	
47	2:1	CRY	1,4-Dioxane	Amorphous	
48	2:1	CRY	N,N-Dimethylacetamide	no precipitation	
49	2:1	CRY	N,N-Dimethylformamide	no precipitation	
50	2:1	CRY	Dimethylsulfoxide	no precipitation	
51	1:1	CRY	Ethanol	KET-LYS P1	
52	1:1	CRY	Methanol	KET-LYS P1	
53	1:1	CRY	Tetrahydrofuran	no precipitation	
54	1:1	PAI	1. Methanol<br>2. THF	no precipitation	
55	1:1	PAI	1. Ethanol<br>2. THF	no precipitation	
56	1:1	PAI	1. Acetonitrile<br>2. THF	no precipitation	
57	1:1	PAI	1. Methanol<br>2. Ethyl Acetate	KET-LYS P2	Selected procedure for P2
58	1:1	PAI	1. 2-Propanol<br>2. Acetonitrile	Amorphous	
59	1:1	PAI	1. Ethanol<br>2. Acetonitrile	KET-LYS P2 + KET	
60	1:1	PAI	1. Ethanol<br>2. Ethyl Acetate	KET-LYS P2	Low yield

GR: grinding; KN: kneading; Ev: Evaporation; SL: Slurry; PAD: Precipitation by antisolvent addition to a supersaturated solution. A solution of KET in selected solvent was added dropwise to aqueous solution of LYS, the solvent becomes antisolvent for the species KET-LYS P1.
PAI: Precipitation by supersatured solution addition to the antisolvent. A solution of KET and LYS in solvent 1 was added to the antisolvent 2.
CRY: The experiments were performed by adding a saturated solution of Ketoprofen to solid Lysine.
LT: Low Temperature (5-8 °C); RT: Room Temperature (20-25 °C); HT: High Temperature (60 °C).

Table S2. Tested samples and model taste solutions for E-tongue analysis.

Compound	Composition	Concentration
KET-LYS P1	Ketoprofen:Lysine 1:1	40 mg/20 mL
KET-LYS P2	Ketoprofen:Lysine 1:1	40 mg/20 mL
Sweet	Fructose	33 mg/30 mL (0.006 mol/L)
Bitter	$MgCl_2$	2.7 mg/30 mL (0.001 mol/L)
Salty	NaCl	1.66 mg/30 mL (0.001 mol/L)

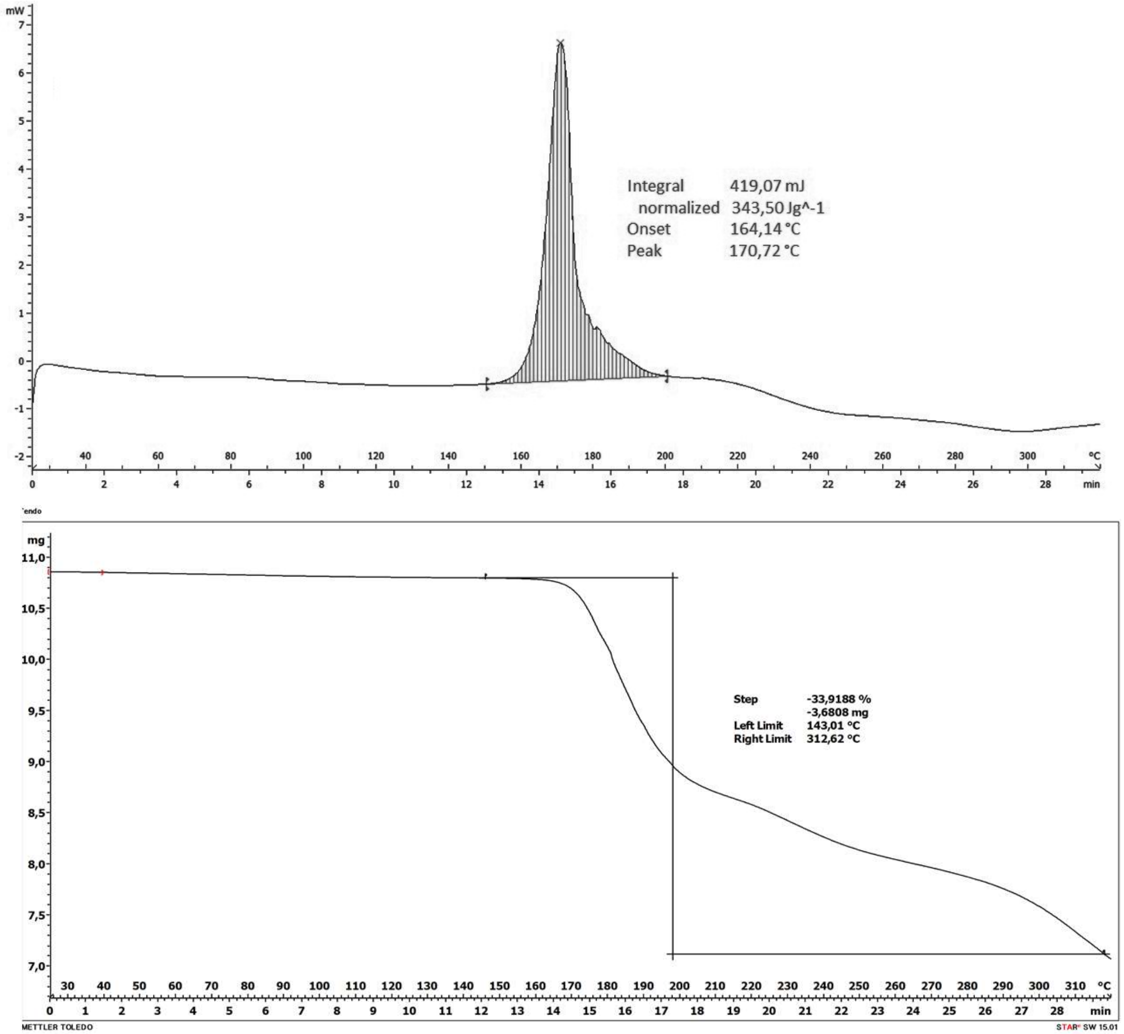

Figure S1. DSC and TGA of KET-LYS P1. KET-LYS P1 DSC profile (upper panel) showed an endothermic event occurring at 170.7 °C (onset 164.1 °C), while TGA analysis (lower panel) showed compound degradation.

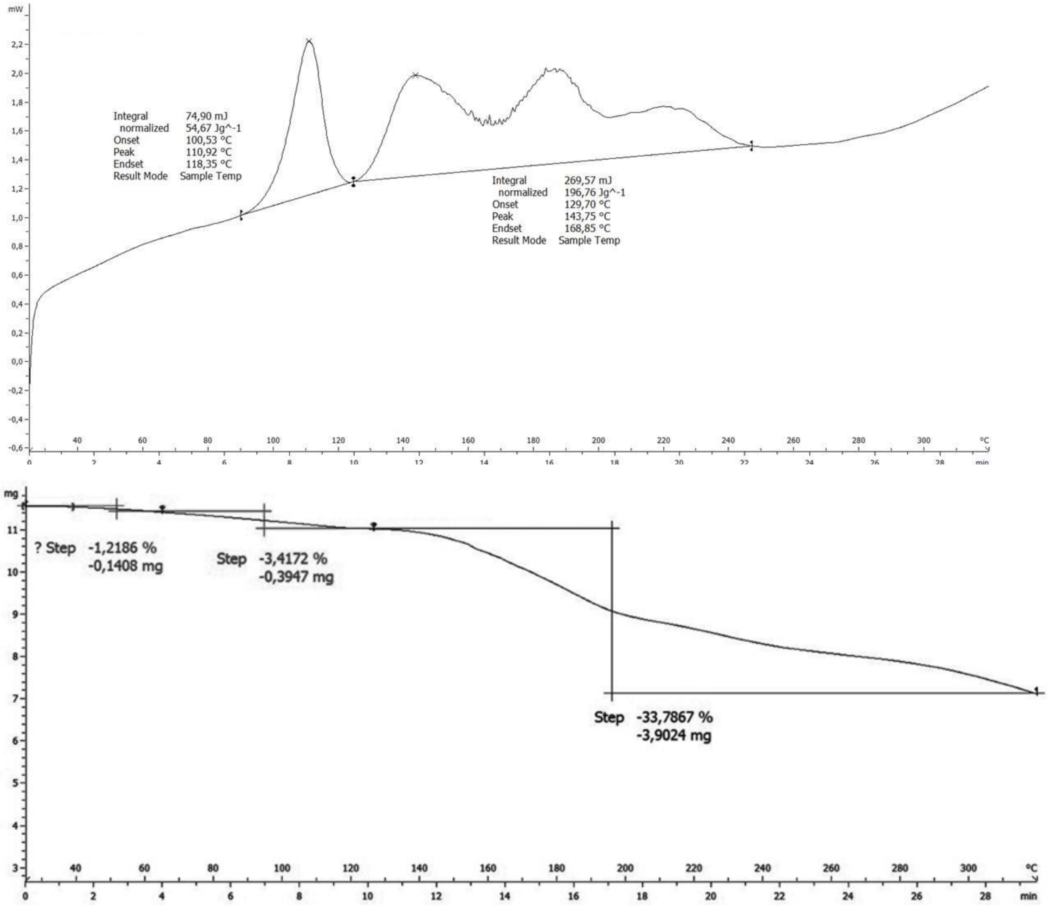

Figure S2. DSC and TGA of KET-LYS P2. Multiple endothermic peaks are detectable in KET-LYS P2 DSC profile (upper panel), the first one occurring at 110.9 °C (onset 100.5°C), while the other multiple partially overlapped endothermic peaks at above 120°C. Progressive degradation of the compound is visible in TGA analysis (lower panel).

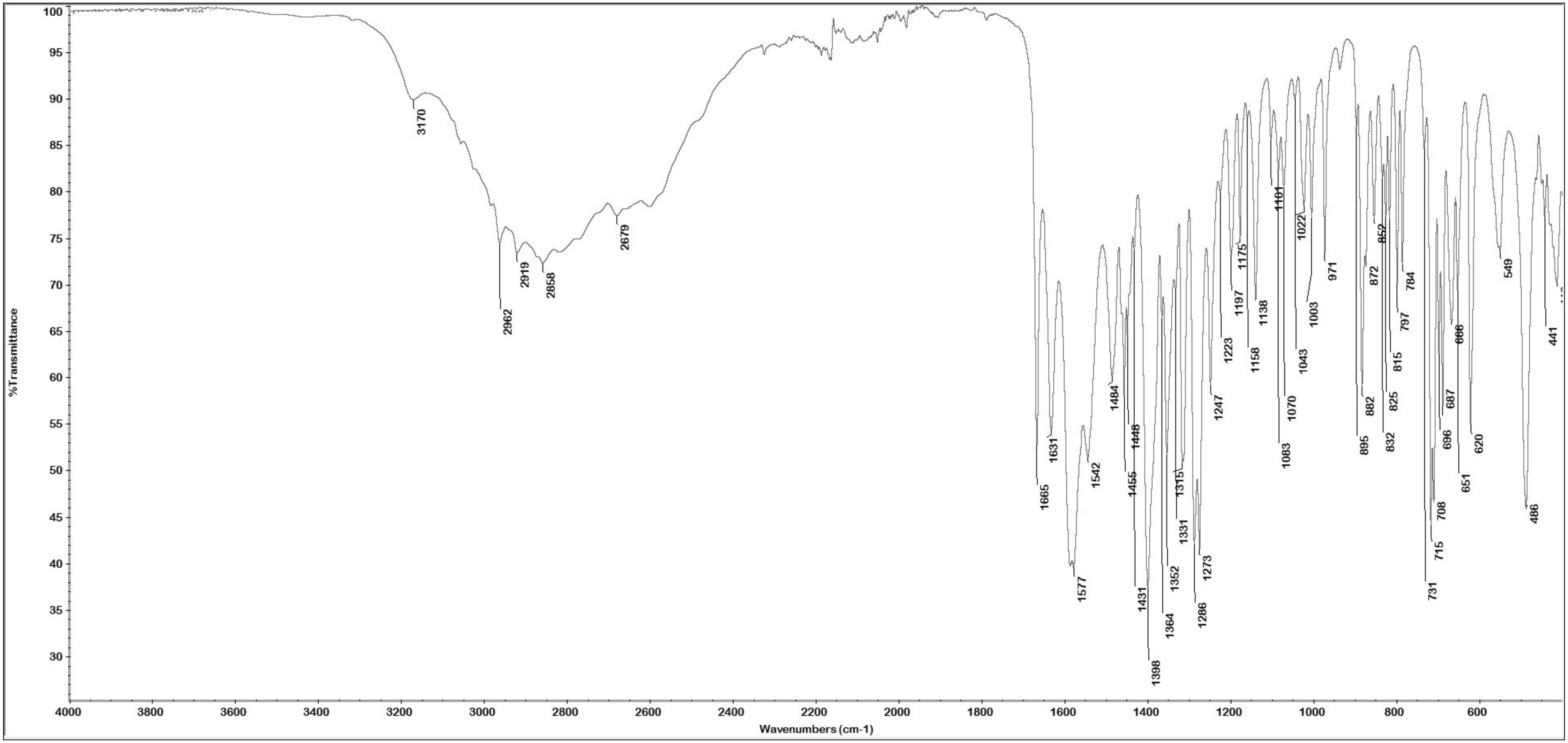

Figure S3. FT-IR of KET-LYS P1. The IR band was centered around 3160 cm^{-1}.

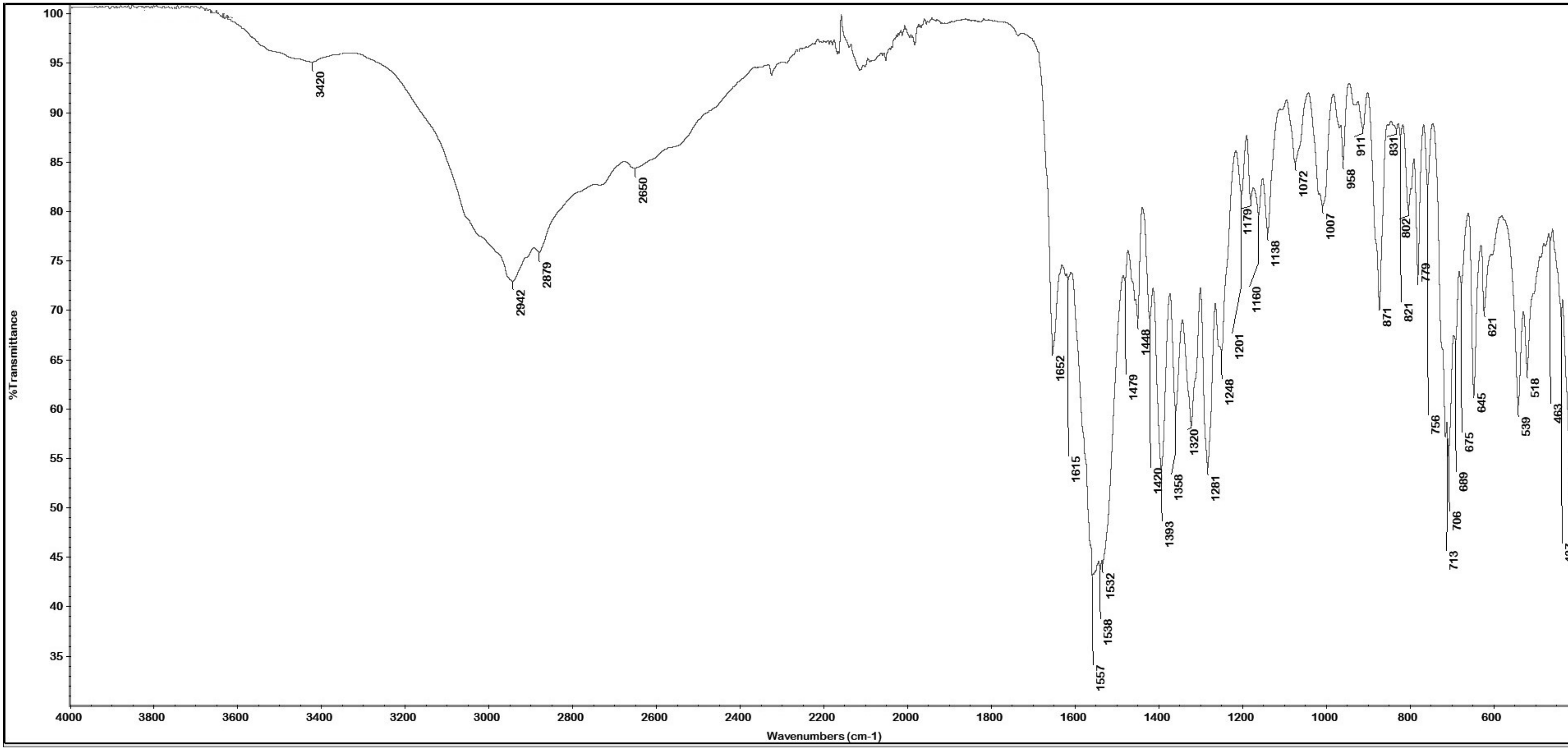

Figure S4. FT-IR of KET-LYS P2. A very broad band is detectable at 3400 - 3660 cm^{-1}.

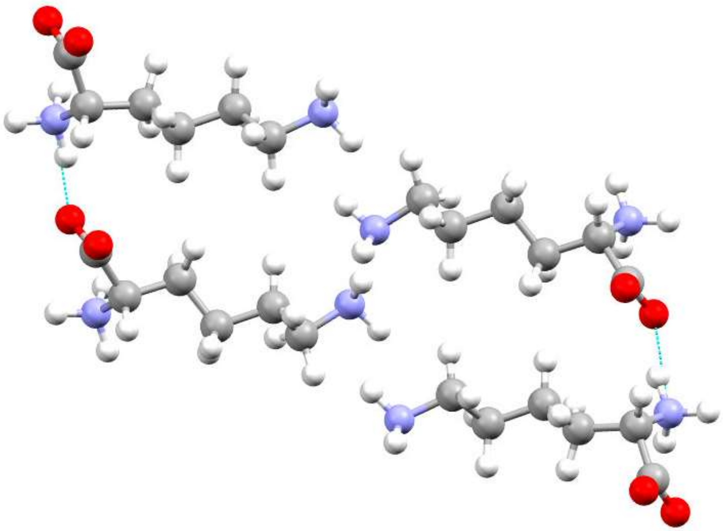

Figure S5. Crystal structure of L-lysine. C = grey; O = red; N = azure; H = white; hydrogen bonds are indicated through cyan dotted lines.

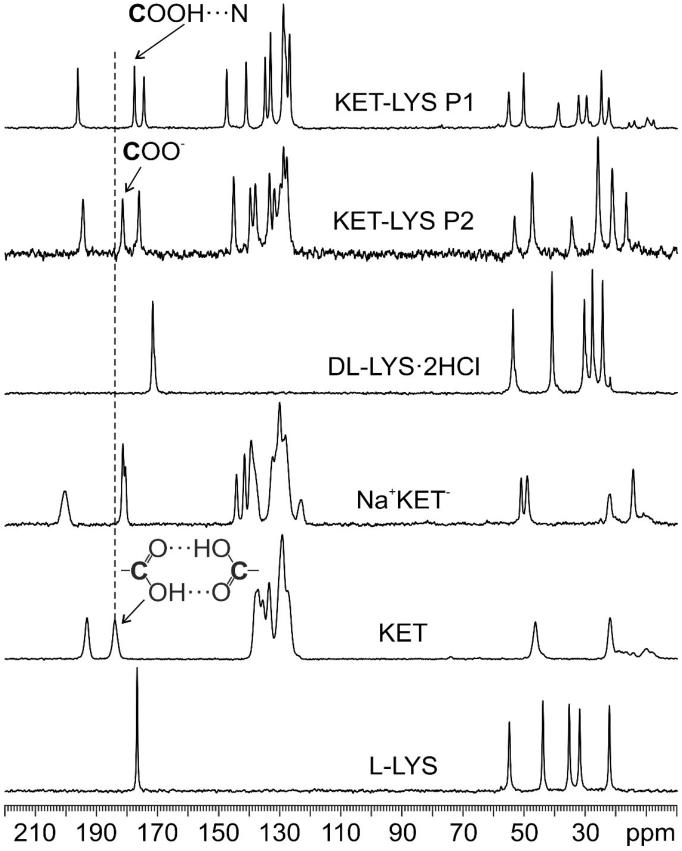

Figure S6. ^{13}C CPMAS spectra of samples KET, L-LYS, $Na^{+}KET^{-}$, DL-LYS·2HCl, KET-LYS P1 and KET-LYS P2. Depending on the employed instrument, the resonance frequency for ^{13}C equals 100 or 150 MHz, while the spinning speed is 12 or 20 kHz, respectively (see Material and Methods for details). All the spectra were acquired at room temperature, except for KET-LYS P2, which was acquired at 273 K. The labels are referred to the carboxylic group of KET.

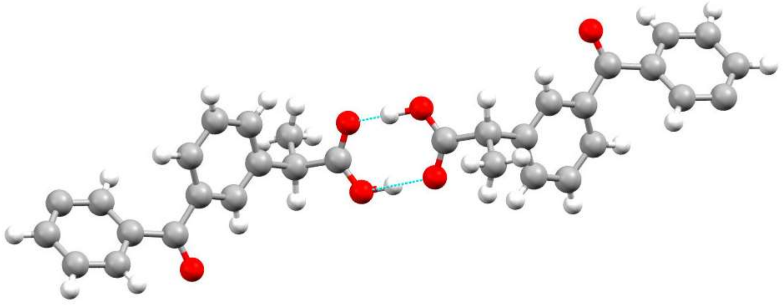

Figure S7. Crystal structure of (R,S)-KET displaying the typical carboxylic homodimeric synthon. C = grey; O = red; H = white; hydrogen bonds are indicated through cyan dotted lines.

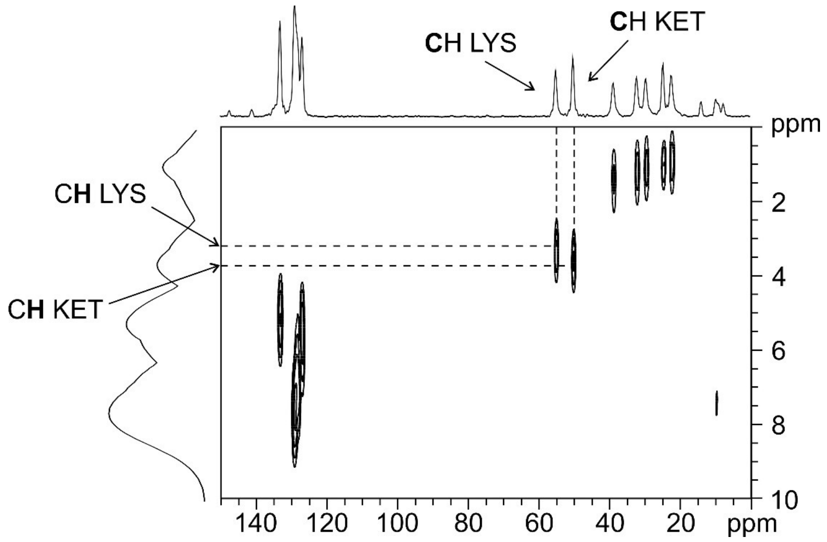

Figure S8. On-resonance ^{1}H-^{13}C FSLG HETCOR spectrum (contact time = 0.1 ms) of KET-LYS P1. Above, ^{13}C spectrum; on the left, ^{1}H spectrum. Dashed lines represent significant correlations among covalently bonded protons and C atoms in the crystal structure (see main text). Spinning speed = 12 kHz, room temperature.

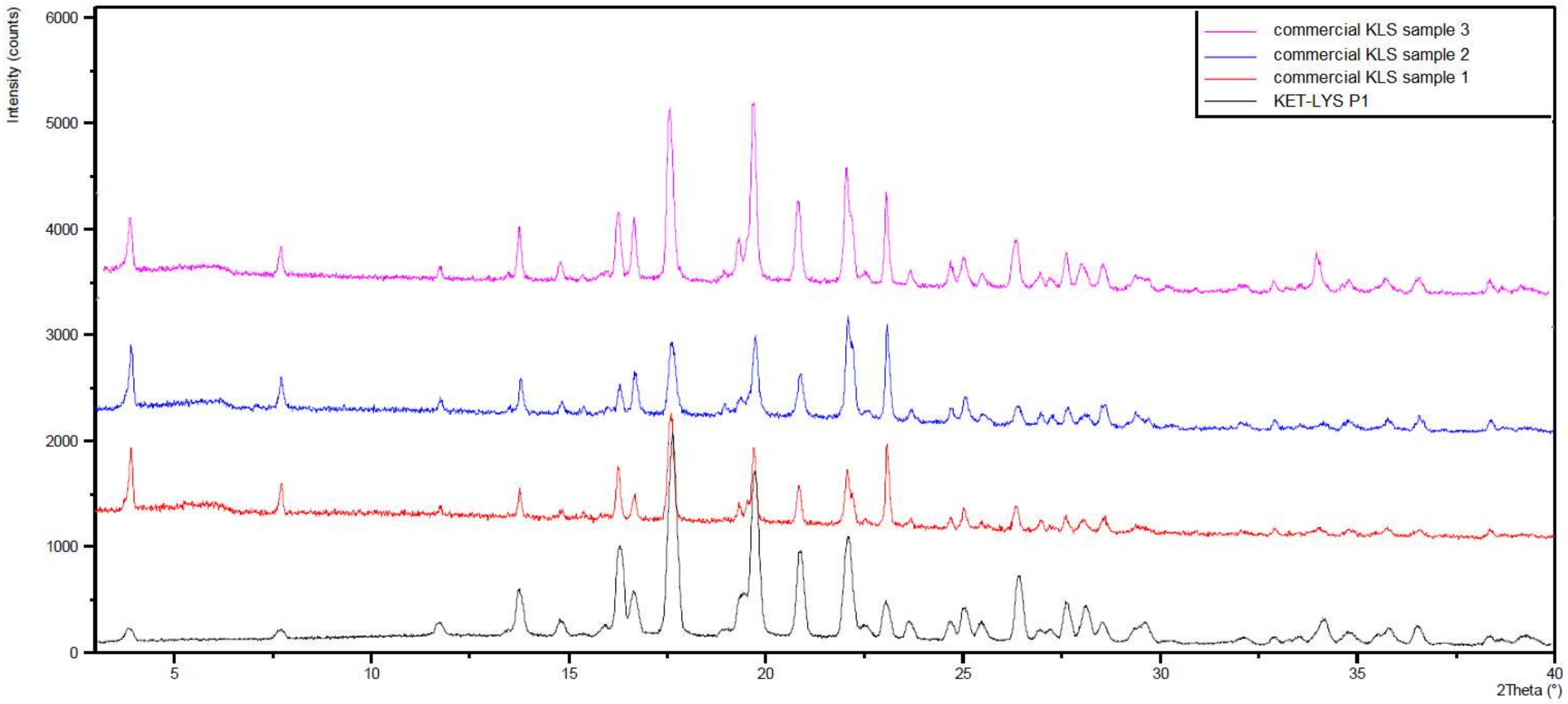

Figure S9. XRPD comparison between KET-LYS P1 and commercial KLS samples. The diffraction patterns of KET-LYS P1 and commercial KLS are superimposable.
